# Assessment of shoulder position variation and its impact on IMRT and VMAT doses for head and neck cancer

**DOI:** 10.1186/1748-717X-7-19

**Published:** 2012-02-08

**Authors:** Emily Neubauer, Lei Dong, David S Followill, Adam S Garden, Laurence E Court, R Allen White, Stephen F Kry

**Affiliations:** 1 Department of Radiation Physics, The University of Texas MD Anderson Cancer Center, 1515 Holcombe Blvd. Houston, TX, 77030 USA; 2 Department of Radiation Oncology, The University of Texas MD Anderson Cancer Center, 1515 Holcombe Blvd. Houston, TX, 77030 USA; 3 Department of Bioinformatics and Computational Biology, The University of Texas MD Anderson Cancer Center, 1515 Holcombe Blvd. Houston, TX, 77030 USA

**Keywords:** Head and Neck, shoulder shifts, IMRT, VMAT, setup, shoulder

## Abstract

**Background:**

For radiotherapy of the head and neck, 5-point mask immobilization is used to stabilize the shoulders. Still, the daily position of the shoulders during treatment may be different from the position in the treatment plan despite correct isocenter setup. The purpose of this study was to determine the interfractional displacement of the shoulders relative to isocenter over the course of treatment and the associated dosimetric effect of this displacement.

**Methods:**

The extent of shoulder displacements relative to isocenter was assessed for 10 patients in 5-point thermoplastic masks using image registration and daily CT-on-rails scans. Dosimetric effects on IMRT and VMAT plans were evaluated in Pinnacle based on simulation CTs modified to represent shoulder shifts between 3 and 15 mm in the superior-inferior, anterior-posterior, and right-left directions. The impact of clinically observed shoulder shifts on the low-neck dose distributions was examined.

**Results:**

Shoulder motion was 2-5 mm in each direction on average but reached 20 mm. Superior shifts resulted in coverage loss, whereas inferior shifts increased the dose to the brachial plexus. These findings were generally consistent for both IMRT and VMAT plans. Over a course of observed shifts, the dose to 99% of the CTV decreased by up to 101 cGy, and the brachial plexus dose increased by up to 72 cGy.

**Conclusions:**

he position of the shoulder affects target coverage and critical structure dose, and may therefore be a concern during the setup of head and neck patients, particularly those with low neck primary disease.

## Background

Patient positioning and immobilization are essential in radiation therapy. Although extensive effort is spent in positioning and immobilizing the patient, the focus is on target alignment; the position of the body away from isocenter is often ignored. Nevertheless, such distant body positions may affect the delivered dose distribution.

For head and neck radiotherapy or other treatments involving the low neck, the position of the shoulders is of particular concern. In many cases, 5-point masks that cover the head and shoulders are used to immobilize the patient. Still, without any displacement of isocenter, the shoulders can be in a position different from the one in the treatment plan. Most IMRT treatments are delivered in a co-planar beam arrangement, so if the patient's shoulders move superior relative to the planning setup the shoulders could intercept the radiation beams and cause an underdosing of the tumor. Conversely, inferior shifts could increase the dose to critical structures. This impact is of particular concern in treatments that have segments near or through the shoulders, as is often the case with intensity-modulated radiation therapy (IMRT). The issue is also important with volumetric modulated arc therapy (VMAT), where the gantry travels in an arc around the patient and the beam could routinely pass through the shoulders [1,2].

Previous studies have found shoulder displacement (in conjunction with isocenter setup) in excess of 1 cm [3,4]. However, these shoulder displacements were assessed by setup images or port films, which offer only 2-dimensional information. Furthermore, although the dosimetric impact of these shoulder shifts was speculated upon, it was not quantified.

Therefore, this study sought to determine the following: first, the extent of 3-dimensional shoulder motion relative to treatment isocenter associated with radiotherapy of the head and neck; second, the dosimetric effect of shoulder position variability on IMRT and VMAT treatments.

## Materials and methods

### Shoulder displacements

Daily shoulder position variation relative to treatment isocenter was quantified using computed tomography (CT)-based bony alignment for 10 patients with lower neck disease involvement. These patients were diagnosed with cancers of the nasopharynx, oropharynx, spine, and mouth, and were treated with 40-70 Gy to the primary tumors with simultaneous boosts to the nodes if needed. All of the patients were simulated and immobilized daily with a 5-point thermoplastic mask (Orfit Industries, Belgium) which fits over the head and shoulders. In addition to the 5-point mask, three of the patients were also simulated with wrist straps that pulled the shoulders inferiorly. Two of these patients with shoulder pulls were also treated with the straps in place. Shoulder variation was evaluated with daily CT scans from an in-room CT-on-rails scanner. The CT on rails had a large field of view (50 cm), allowing both humeral heads to be captured in the daily image.

Shoulder position variations were determined relative to the planning image. Each humeral head and the clinical bony alignment structure (the C2 vertebrae for 7 patients, C3 for 1 patient, C1-C3 for one patient, and C7-T3 for 1 patient) were contoured on the planning CT,. In-house image registration software [5,6] was used to locate the humeral heads and the alignment structure on each of the daily CTs. The results were verified visually. To quantify shoulder motion, the centroid coordinates for each humeral head were determined relative to the alignment structure, yielding the daily difference in each shoulder position in each direction relative to isocenter.

To determine any trends in shoulder position with time, a linear regression was performed on the displacement versus fraction. Significance was determined by a p-value less than 0.05 and the 95% confidence interval not including 0.

### Dosimetric impact

To investigate the dosimetric impact of shoulder shifts, an IMRT and a SmartArc plan were developed on the simulation CT for 3 head and neck patients using the Pinnacle treatment planning system (Pinnacle^3 ^version 9, Fitchburg, WI). Patient 1 had a base of tongue tumor treated to 60 Gy in 30 fractions with a bilateral simultaneous integrated boost to two nodal volumes: one to 57 Gy and another to 54 Gy, and was treated with 10 coplanar beams (200°, 240°, 280°, 320°, 0°, 40°, 80°, 120°,160°, and 180°). Patient 2 had an unknown primary treated to 69.96 Gy in 33 fractions, with a unilateral simultaneous integrated boost to two nodal volumes: one to 65 Gy and one to 63 Gy, and was treated with 7 fields (350°, 20°, 55°, 85°, 120°, 150°, and 175°). Patient 3 had a laryngeal primary treated to 60 Gy in 30 fractions with a simultaneous integrated boost to bilateral nodes to 54 Gy, and was treated with 9 fields (200°, 240°, 280°, 320°, 0°, 40°, 80°, 120°, and 160°). The VMAT plans for all 3 patients used two 360° arcs. The IMRT and VMAT fields extended into the lower neck. Patients treated with a half-beam matched technique (using AP fields to treat the low neck) were not examined because the dose disturbance due to shoulder variability was assumed to be less. Per clinical practice, IMRT beams that directly intersected the shoulders from the lateral direction had their inferior border raised to avoid shoulder penetration (this included the 85° beam on Patient 2 only). All plans met MD Anderson Cancer Center clinical planning objectives.

**Figure 1:**
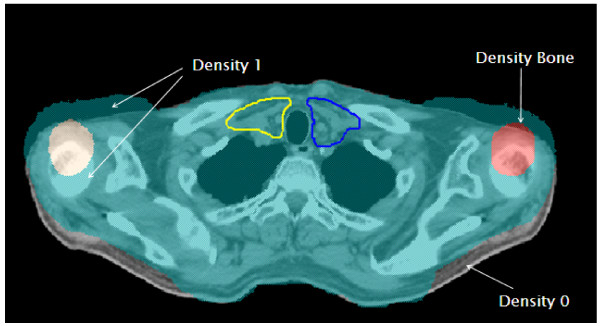
**15 mm anterior shift**. Body contours are shifted, and the appropriate density is set.

The CT images were then manually edited in Pinnacle to simulate shoulder shifts on each patient of 3, 5, and 15 mm in the superior and inferior (SI) directions, 3 and 15 mm in the anterior and posterior (AP) directions, and 15 mm in the right or left (RL) direction. These shifts were realized by adjusting the body and bony contours of the shoulders. The medial areas of the chest and back remained in the same location as the original scans. The relative density of tissues was forced to 1 or 0 as necessary to compensate for tissue moving into or out of the new location (Figure 1); similarly, the average density of each shifted bone was maintained. The baseline plans were then recalculated on each of these adjusted images (referred to as "shifted plans").

This method of manual CT editing allowed for isolation of specific shoulder shifts and excluded the effects of other anatomical differences and internal motion, which was not available on the daily CT data. The validity of the manual CT edits was verified as follows. A patient was identified who, on subsequent CTs (taken on the same day), showed a 1 cm superior shift in both shoulders and a 3.6 mm anterior shift of one shoulder. The first CT was also edited by hand to mimic the second CT. The dose distributions for the daily CT with shifted shoulders (second scan) was compared to the dose distributions for the manually edited CT, and these were found to be nearly identical. Therefore, the manually edited images gave a reasonable estimation of the impact of the shoulder shifts.

### Clinical impact

The dosimetric impact determined above describes the impact of a single shift occurring every day, rather than the dosimetric impact of a series of shifts that could be encountered during routine clinical treatment. To assess the clinical impact, the target coverage and critical structure dose changes to clinical IMRT plans associated with each shoulder shift were combined with a set of shifts observed over the course of treatment. The cumulative changes in dose to a representative point chosen in the clinical target volume (CTV), 99% of the CTV (D99%), and to critical structures due to shoulder variation were estimated. Specifically, for each shift in the dosimetric studies, the change in point dose, D99%, and maximum dose to 0.1 cm^3 ^of the brachial plexus per fraction was multiplied by the number of fractions for which each shift was observed.

## Results

### Observed shifts

**Figure 2:**
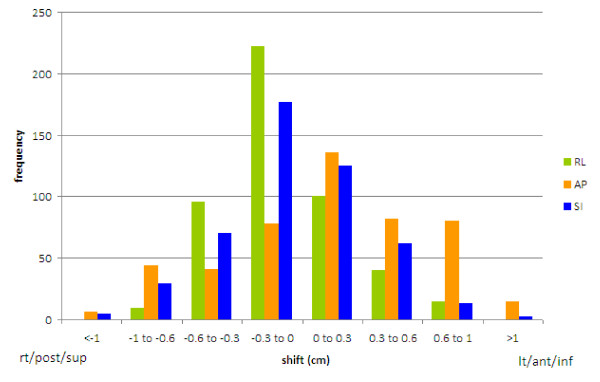
**Total distribution of shifts in all 3 directions**. 85% were less than 0.6 cm. Shifts greater than 1 cm, were seen in the superior-inferior (SI) and anterior-posterior (AP) directions. RL = right-left.

**Figure 3:**
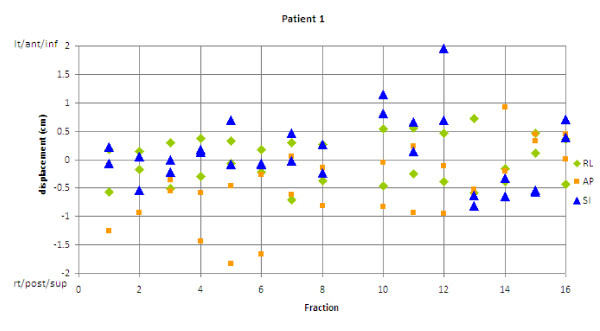
**Shoulder displacement vs. treatment fraction**. Patient with large variation in shoulder displacements over treatment. AP = anterior-posterior; RL = right-left; SI = superior-inferior.

**Figure 4:**
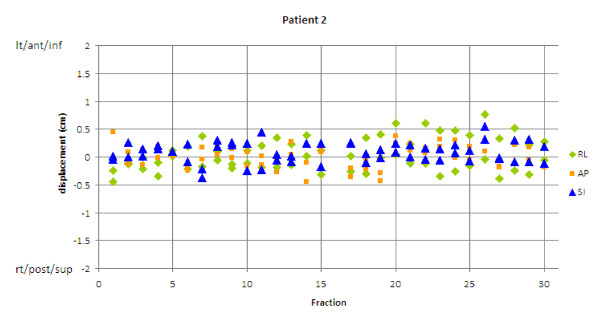
**Shoulder displacement vs. treatment fraction**. Patient with small variation in shoulder displacements over treatment. AP = anterior-posterior; RL = right-left; SI = superior-inferior.

Ten patients and 243 CTs were examined. The average shoulder shifts observed were 2-6 mm (Table 1). Most (85%) of the observed shifts were less than 6 mm, but 2% of shifts were greater than 10 mm, and all patients had at least 1 shift greater than 5 mm. Shifts greater than 10 mm were observed in all directions except RL (Figure 2). Of note, 4 of the 10 patients had their maximum shifts occur in the superior direction. Some patients showed very irregular shoulder positioning (Figure 3), whereas others showed very stable positioning (Figure 4). Most patients had a combination of random and systematic shifts; however, large shifts tended to be random. Patients with wrist straps that pulled the shoulders inferiorly did not show consistently smaller shifts than those treated without. For example, the patient in Figure 3 did have shoulder pulls for each treatment, and the patient shown in Figure 4 did not.

**Table 1:** Range and average magnitude of shifts (cm) in each direction for the right and left shoulder and an average net displacment determined by the 3D-vector of each shoulder.

	RL	AP	SI	Net
Range	0-0.85	0-1.83	0-1.96	0.10-2.01
Average (Right shoulder)	0.22	0.36	0.35	0.55
Average (Left shoulder)	0.26	0.48	0.27	0.60
Average net				0.58

AP = anterior-posterior; RL = right-left; SI = superior-inferior

While it might be expected that the size of shoulder displacements increase with time (as the patient loses weight and the mask gets looser), this was not observed. Regression analysis of the magnitude of the shoulder shift over time showed that the vast majority of patients did not demonstrate an increase in the size of the shoulder shift with time (p > 0.05) in any of the RL, AP, or SI directions, nor did they demonstrate an increase in the magnitude of 3-dimensional displacement. At most 3 patients showed a statistical increase or decrease in magnitude of shoulder displacement in any given direction (p < 0.05), however, these were equally increases and decreases in magnitude.

### Dosimetric impact

The target coverage losses for 2 CTV dose levels in the neck are presented in Table 2 for shoulder shifts that induced a loss of coverage. For both IMRT and VMAT plans, shoulder shifts in the superior direction resulted in the greatest loss of coverage, and the loss was comparable between the two modalities. Larger shifts resulted in larger coverage losses. While 5 mm superior shifts caused coverage losses of 2-24 cm^3 ^at the 100% dose level, 15 mm superior shifts could cause coverage losses of more than 100 cm^3 ^at the 100% dose level and more than 40 cm^3 ^at the 95% dose level. The corresponding percent loss of target coverage is shown for patient 1 in Table 3. Target coverage losses in the 100% isodose line were seen around C6-C7, and were between 7% and 78% of the tumor volume located at these vertebral levels.

**Table 2:** Lower neck target dose changes (cm^3^) due to important shifts.

Shift		Patient 1			Patient 2			Patient 3		
		
		V100%	V98%	V95%	V100%	V98%	V95%	V100%	V98%	V95%
IMRT										
3 mm superior	Target 1	-4	0	0	-4	0	0	0	0	0
	Target 2	0	0	0	-1	0	0	-1	0	0
5 mm superior	Target 1	-19	-1	0	-14	-2	0	-2	0	0
	Target 2	-3	0	0	-9	-1	0	-7	0	0
15 mm superior	Target 1	-132	-73	-9	-53	-31	-4	-58	-20	-2
	Target 2	-29	-5	-1	-64	-35	-12	-77	-25	-7
15 mm posterior	Target 1	-12	0	0	-8	0	0	-4	-1	0
	Target 2	-5	-1	0	-7	-2	-1	-25	-4	-1
VMAT										
3 mm superior	Target 1	-4	0	0	-2	-2	0	0	0	0
	Target 2	-2	0	0	0	0	0	-1	0	0
5 mm superior	Target 1	-24	-3	0	-7	-4	0	0	0	0
	Target 2	-10	-1	0	-5	0	0	-6	-1	0
15 mm superior	Target 1	-152	-102	-41	-36	-13	-1	-49	-7	0
	Target 2	-43	-22	-10	-36	-11	0	-65	-29	-7
15 mm posterior	Target 1	-1	0	0	-1	-1	0	0	0	0
	Target 2	0	0	0	-1	-1	-1	-3	-2	-1

Target 1 = higher dose clinical target volume (CTV); target 2 = lower dose CTV; V100%, V98% and V95% are the volumes covered by the 100%, 98%, and 95% isodose lines, respectively; 15 mm superior shifts show greatest loss of coverage for both IMRT and VMAT, and 15 mm posterior shifts show loss of coverage greater than 4 cm^3 ^for IMRT plans only.

**Table 3:** Target coverage in the C6-C7 region

	IMRT			VMAT		
	
	100%	98%	95%	100%	98%	95%
C6-C7						
No shift	97	98	100	94	97	99
5 mm superior	90	98	100	84	96	99
15 mm superior	23	53	94	16	35	72
C7-T2						
No shift	98	100	100	--	--	--
15 mm posterior	89	99	100	--	--	--

Percentage of the clinical target volume (CTV) in the C6-C7 region covered by the 100%, 98%, and 95% isodose lines with no shift and with superior shifts for IMRT and VMAT plans, as well as the percent coverage of the CTV in the C7-T2 region with no shift and a 15 mm posterior shift. All percentages were evaluated for Patient 1.

For IMRT plans (but not VMAT plans), large posterior shifts also caused loss of coverage (Table 2). Coverage loss was seen in the C7-T2 region, and was up to 11% of the local target volume (Table 3). Shifts in other directions (as well as smaller posterior shifts) did not cause a change in target coverage.

Dose elevation to critical structures associated with shoulder shifts is presented in Table 4. In general, dose increases to the spinal cord were less than 40 cGy for any shoulder shift considered. However, the dose to the brachial plexus did increase, most substantially with inferior shifts. This was seen for both IMRT and VMAT plans, and doses could be elevated by 410 cGy for 15 mm inferior shifts. For IMRT plans (but not VMAT plans), an increase in brachial plexus dose was also found with anterior shifts.

**Table 4:** Brachial plexus dose change (cm^3^) due to important shifts.

	Patient 1		Patient 2		Patient 3	
	
Shift	IMRT	VMAT	IMRT	VMAT	IMRT	VMAT
3 mm inferior						
Max DVH dose (cGy)	-1	-3	116	12	45	-29
Max 0.1 cm^3 ^(cGy)	0	40	100	45	40	60
V60 (cm^3^)	0	0	0	0	0	0
15 mm inferior						
Max DVH dose (cGy)	49	372	203	186	218	259
Max 0.1 cm^3 ^(cGy)	60	410	205	195	210	240
V60 (cm^3^)	1	3	0	0	0	1
3 mm anterior						
Max DVH dose (cGy)	-1	0	22	1	23	-14
Max 0.1 cm^3 ^(cGy)	0	0	15	5	20	-20
V60 (cm^3^)	0	0	0	0	0	0
15 mm anterior						
Max DVH dose (cGy)	-1	1	125	10	109	-64
Max 0.1 cm^3 ^(cGy)	0	0	100	0	100	-70
V60 (cm^3^)	0	0	0	0	0	0

The changes in maximum dose displayed on the dose volume histogram (DVH), maximum dose to 0.1 cm^3^, and volume receiving 60 Gy (V60) are shown. Inferior shifts show the greatest increase in dose to the brachial plexus for both intensity-modulated radiation therapy (IMRT) and volumetric modulated arc therapy (VMAT) plans.

### Clinical impact

When a set of observed shifts (from the patient shown in Figure 3: one 3 mm superior, four 5 mm superior, and seven 15 mm posterior shifts) was applied to the 3 dosimetric studies, the greatest loss of dose to 99% of the lower neck CTV of any of the cases was a decrease by 101 cGy (range, 14-101 cGy), with a corresponding point dose decrease of 57-95 cGy. The largest observed losses to 99% of the CTV per fraction (based on a 2 Gy fraction) are outlined in Table 5. Table 5 also shows how frequently certain shifts must occur to lose 1, 2, or 3 Gy to 99% of the CTV; typically, large numbers of fractions are required. The brachial plexus dose was similarly evaluated for the six 5 mm inferior and two 15 mm inferior shifts that were observed. The largest increase in dose to 0.1 cm^3 ^of the brachial plexus of all of the examined dosimetric cases was 72 cGy (range, 64-72 cGy).

**Table 5:** Dose loss due to shoulder shifts

		Frequency for losses of:		
		
Shift	Dose lost per fraction (cGy)	1 Gy	2 Gy	3 Gy
5 mm superior	-3	33	--	--
15 mm superior	-11	9	18	27
15 mm posterior	-5	20	--	--
5 mm superior + 15 mm posterior	-8	13	25	--
15 mm superior + 15 mm posterior	-16	6	13	19

Dose lost to 99% of the clinical target volume due to superior and posterior shifts and the number of shifts required for a D99% loss of 1, 2, or 3 Gy. Dashes represent > 35 fractions required.

## Discussion

This study found that large shifts (> 1 cm) routinely occurred during the course of radiotherapy, even when 5-point masks are used. In general, there was no trend with time in the magnitude of shifts in the RL, AP, or SI directions. Although some patients did show either an increasing or decreasing trend in the size of shoulder shift as treatment progressed, equal numbers of patients had shifts that were larger or smaller and the vast majority had no trend in any direction. While large shoulder shifts could be expected to occur late in treatment due to patient relaxation or weight loss, we observed such shifts to occur equally both early and late in treatment.

In this study, we observed the largest shoulder shifts in the AP and SI directions, which is different from results reported in the literature where the largest shifts were found in the RL direction. However, our study examined the position after correct isocenter setup, whereas the others examined the position prior to correct setup [3,4] and evaluated how much the shoulders or the shoulder region had to be moved to properly align isocenter. These studies were also limited by visibility on megavoltage images. However, the magnitudes of the shoulder displacements were comparable between our study and the others, being typically less than 6 mm.

The observed shoulder motion is consistent with previous studies that showed lower-neck structures experience more setup variability when aligning to C2 [7,8]. If the patient were setup using a lower target for alignment, such as C7, there may be a reduction in the size of the observed shoulder shift as medial lower-neck targets would show less variability. However, this was not observed in this study. Only one patient was aligned to vertebral bodies in the low neck (C7-T3) and this patient showed a great deal of shoulder variability (Figure 3). The authors believe that the shoulders may still show large displacements because the shoulders are far from mid-line and can move independently of the low neck vertebral bodies, and are often ignored in setup.

One drawback to our method of aligning the patient is that is only occurs in the 3 linear directions without any rotational component. Therefore, it was not possible to reduce or minimize shoulder shifts that were the result of rotated anatomy. Therefore, the size of observed shoulder shifts may be reduced if the IGRT process includes rotational as well as translational shifts. However, based on the findings throughout this work that the shoulders move largely independently, it is likely that large shoulder displacements could still occur, even with rotation accounted for.

The greatest change in lower neck target coverage was found for superior shifts because these brought shoulder tissue into a region where it was previously absent, thereby changing the depth and beam attenuation to the target. This was observed even when the inferior jaws of lateral fields are closed above the shoulder, as seen in Patient 2 (Table 2). A similar result was found for large posterior shifts, which resulted in target coverage loss for IMRT plans. VMAT plans did not show a similar loss of coverage for posterior shifts. This likely resulted from the Monitor Unit (MU) distribution; even though beams were evenly spaced around the patient, the IMRT plans had ~50% of the MU from posterior or posterior oblique beams. In contrast, VMAT plans had a relatively even distribution of MUs with gantry angle.

It is important to note that the coverage loss from superior and posterior shifts was not compensated for by an equivalent increase in coverage from inferior or anterior shifts. That is, the effect of the shift does not average out over the course of treatment. This can be understood by considering that a superior shift will cause attenuation and loss of coverage to a transverse section of the neck. A subsequent inferior shift will increase the dose slightly, but to a different transverse section of the neck (an inferior section), thereby not compensating for the dose loss associated with the superior shift. The position of the shoulder each day has an impact on coverage, and a mean shoulder position will not represent the total effect of the shoulder movement over the course of treatment. Documentation of the number of superior and large posterior shifts will give the best information about loss of coverage to the target.

In a clinical setting, the most important impact of shoulder motion is the loss of target coverage (~1 Gy to 99% of a lower neck target). This may be important, particularly if there is primary disease in that region. Moreover, shoulder shifts may be an important consideration for Stereotactic Spine Radiosurgery (SSRS) or for other patients undergoing hypofractionated therapy with lesions near C6. These IMRT plans are delivered in few (or one) fractions, so large errors are not mitigated by subsequent fractions. It is important to consider that this positioning study found no trend with time for large shoulder shifts; large shifts (> 1 cm) were seen in the first few days of treatment for many patients. In addition to these considerations, treatments that use predominantly posterior beams may suffer coverage loss worse than that predicted in this study. Also, systematic shoulder shifts are more likely to cause substantial dose losses similar to those shown in Table 2. While most patients in this study had small systematic shifts, 2 patients demonstrated large (8-10 mm) systematic shifts.

When we evaluated dosimetric impact, no clinically important change was seen in dose to the spinal cord because it had been avoided in the treatment plans and it was always associated with low photon fluences. However, the brachial plexus was located close to the targets, so changes in shoulder position affected beam attenuation and dose to this structure. The overall 72 cGy increase in dose to 0.1 cm^3 ^of the brachial plexus is not likely to cause harm because the max dose to 0.1 cm^3 ^of the brachial plexus is not always in the same location within the structure, depending on shoulder position. The daily increase in dose to 0.1 cm^3 ^of the brachial plexus was a few cGy; therefore, the dose escalation required to receive a TD5/5 dose of over 60 Gy [9] on a single day was not observed.

## Conclusions

In general, shoulder positional variation is approximately 2-6 mm; however, large shifts up to 2 cm may be possible even when 5-point masks that extend over the shoulders are used. Large superior shifts can lead to underdosing of lower neck targets if they occur frequently. No trend was seen for large shifts with time, so patients are at risk for having large shoulder displacements early and late in treatment. Because up to 1 Gy can be lost from shoulder variation over the course of treatment, including shoulder position in daily setup procedures would be beneficial to head and neck patients with low neck targets. The dose-coverage losses demonstrated in this study may underestimate losses that occur for hypofractionated or single fraction treatments; therefore, the position of the shoulder warrants particular attention in these instances. If they can be captured in the field of view, proper shoulder alignment can be discerned from daily imaging and compared to Digitally Reconstructed Radiographs (DRR). The angle of the clavicle in an AP image can also indicate a superior shoulder shift if it is steeper than it appears in a DRR. If possible, the shoulders or humeral heads can also be used as a secondary alignment point in Cone Beam CT setup. If imaging data is not available due to field of view limitations, indexing the position of shoulders to the treatment couch via mask marks should at least help to avoid superior shifts.

## List of abbreviations

IMRT: Intensity Modulated Radiation Therapy; VMAT: Volume Modulated Arc Therapy; CT: Computed Tomography; RL: Right/Left; AP: Anterior/Posterior; SI: Superior/Inferior; DRR: Digitally Reconstructed Radiograph.

## Competing interests

The authors declare that they have no competing interests.

## Authors' contributions

EN contributed to the design of the study and carried out data collection, analysis and interpretation. LD contributed to the study design and data analysis in Pinnacle. RAW contributed to statistical analysis. ASG contributed to the design of the study and provided patients to analyze. DF contributed to the design of the study and analysis of the data. LEC contributed to the design of the study, interpretation of the data and revision of the manuscript. SFK contributed to the design of the study, data interpretation, and revision of the manuscript. All authors have read and approved the final version of this manuscript.
